# Dielectric dispersion characteristics of the phospholipid bilayer with subnanometer resolution from terahertz to mid-infrared

**DOI:** 10.3389/fbioe.2022.984880

**Published:** 2022-08-31

**Authors:** Ziyi Zhang, Yangmei Li, Zuoxian Xiang, Yindong Huang, Ruixing Wang, Chao Chang

**Affiliations:** ^1^ Innovation Laboratory of Terahertz Biophysics, National Innovation Institute of Defense Technology, Beijing, China; ^2^ School of Physics, Peking University, Beijing, China

**Keywords:** myelinated nerve fiber, phospholipid bilayer, terahertz/mid-infrared, optical/dielectric constants, subnanometer resolution

## Abstract

There is growing interest in whether the myelinated nerve fiber acts as a dielectric waveguide to propagate terahertz to mid-infrared electromagnetic waves, which are presumed stable signal carrier for neurotransmission. The myelin sheath is formed as a multilamellar biomembrane structure, hence insights into the dielectric properties of the phospholipid bilayer is essential for a complete understanding of the myelinated fiber functioning. In this work, by means of atomistic molecular dynamics simulations of the dimyristoylphosphatidylcholine (DMPC) bilayer in water and numerical calculations of carefully layered molecules along with calibration of optical dielectric constants, we for the first time demonstrate the spatially resolved (in sub-nm) dielectric spectrum of the phospholipid bilayer in a remarkably wide range from terahertz to mid-infrared. More specifically, the membrane head regions exhibit both larger real and imaginary permittivities than that of the tail counterparts in the majority of the 1–100 THz band. In addition, the spatial variation of dielectric properties suggests advantageous propagation characteristics of the phospholipid bilayer in a relatively wide band of 55–85 THz, where the electromagnetic waves are well confined within the head regions.

## Introduction

The electromagnetic spectrum from terahertz to mid-infrared region is vital to living organisms since the collective vibrations of most biomacromolecules (e.g., DNA and protein) fall within this frequency range, where many significant physiological phenomena and biomedical applications have been reported (Barone et al., 2005; Kitagawa et al., 2006; Rodrigo et al., 2015; Cheon et al., 2016; Turker-Kaya and Huck, 2017; Mittal et al., 2018; Zhu et al., 2021; Zhang et al., 2021; Li et al., 2021; Li et al., 2022; Sun et al., 2022). In addition, the spectra of the optical constant (refractive index and extinction coefficient), and the dielectric constant (real and imaginary parts of the permittivity) of all biomaterials contain the inherent information of their internal molecules, atoms and chemical bonds, and hence could be utilized as the functional biosignatures (Pethig and Kell, 1987; Parthasarathy et al., 2005; Davidov et al., 2021). A recent experimental study finds that the frog sciatic nerve shows distinct refractive indexes measured at different spots in the terahertz to mid-infrared band, suggesting that the myelinated nerve fiber might act as a decent dielectric waveguide (Liu et al., 2019). Although this finding is a major step forward in supporting that terahertz/mid-infrared electromagnetic waves might be information carriers for neural signal propagation, a question is still left unanswered: what is the specific frequency band for the best information propagation? Intuitively, this band should possess the following features:

1) The band should be continuous and broad. In other words, the frequencies of the electromagnetic waves for the neural signal propagation cannot be a single frequency or some isolated frequencies. This feature contributes to provide a stable and robust communication capability.
2) The refractive index, or the real part of the permittivity, of the myelin sheath should be obviously higher than that of the axon and the extracellular fluid. This feature guarantees that the electromagnetic waves cannot spread to the extracellular fluid and propagate primarily through the myelin sheath rather than the axon.
3) The extinction coefficient, or the imaginary part of the permittivity, of the myelin sheath should be small enough. This feature manifests that the electromagnetic waves can propagate through a considerable long myelin sheath without getting weaker.

In order to answer the above questions, the optical/dielectric properties of the myeline sheath and the axon in the frequency range from terahertz to mid-infrared must be investigated primarily. Researchers have attempted to directly measure the optical/dielectric properties of the myelinated nerve fiber by experiments. Antonov *et al.* carried out the first *in vivo* measurement of the refractive index of a peripheral nerve fiber of the sciatic nerve in *Rana temporaria* using holographic interference microscopy, and the constant refractive indexes of the myelin sheath and the axon in the visible were obtained (Antonov et al., 1983). Rahman *et al.* measured the frequency-dependent refractive index and extinction coefficient of the sciatic nerve of *Xenopus laevis in vitro* for the first time based on a spectrophotometer working in the wavelength range of 860–2,250 nm (Rahman et al., 2018). Tayebi *et al.* analyzed the refractive index dispersion of an individual nerve fiber in striatal medium spiny neurons using a triple-wavelength diffraction phase interferometer (473, 589 and 685 nm) (Tayebi et al., 2019). We find that the related experimental studies are extremely rare due to the limitation of the existing practical techniques and measuring instruments. Currently, to the best of our knowledge, there is no ready-to-use and abundant measured data which could reveal the optical/dielectric properties of the myeline sheath and the axon in the terahertz to mid-infrared spectrum.

It is well known that the myelin sheath and the axon mainly consist of the biomembrane and the intracellular fluid, respectively (Kolb and Whishaw, 1980; Rinholm and Bergersen, 2012; Fields, 2014). As the phospholipid bilayer and the water are respectively the major constituents of the biomembrane and the intracellular fluid, the optical/dielectric properties of the phospholipid bilayer and the water are thus reasonable indicators of those of the myeline sheath and the axon, respectively. The optical/dielectric constants of the water over a wide frequency range from direct current to ultraviolet at various temperature have been thoroughly investigated, and plenty of theoretical and experimental data can be acquired from the available literature (Hale and Querry, 1973; Segelstein 1981; Buchner et al., 1999; Praprotnik and Janežič, 2005; Heyden et al., 2010; Midi et al., 2014; Rowe et al., 2020; Krishnamoorthy et al., 2021). The studies on the biomembrane or phospholipid bilayer also have been started for long. Zhou et al. applied the linear response theory to estimate the susceptibilities across a dilauroylphosphatidylethanolamine (DLPE) bilayer (Zhou and Schulten, 1995). Stern et al. established a rigorous expression to calculate the permittivity of a dipalmitoylphosphatidylcholine (DPPC) by combining statistical mechanics and continuum electrostatics (Stern and Feller, 2003). Tanizaki et al. proposed a three-layered model with different dielectric constants as hydrocarbon, ester group and water based on the generalized Born formalism (Tanizaki and Feig, 2005). Hishida et al. focused on the hydration state of the lipid membrane with techniques of terahertz time-domain spectroscopy and small-angle X-ray scattering, and the dielectric constants of dimyristoylphosphatidylcholine (DMPC) solutions in the frequency range of 0.5–2.6 THz were measured (Hishida and Tanaka, 2011). Hielscher et al. measured the absorbance spectra of six different types of phospholipids in the far-infrared region from 600 to 50 cm^−1^ using Fourier transform infrared spectroscopy (Hielscher and Hellwig, 2012). Siddiquee et al. imaged dioleoylphosphatidylcholine (DOPC) and DPPC membranes based on the local absorption coefficients measured by a scanning near-field optical microscopy system with 640 nm laser beam (Siddiquee et al., 2019). In summary, although previous studies have made great contributions to the frequency-dependent optical/dielectric constants of the phospholipid bilayer, however, the dispersion profiles obtained by both theoretical and experiment methods are almost narrowband spectra. In addition, it can be found that the studies of the subnanometer resolution optical/dielectric properties of the phospholipid bilayer are only focused on the static permittivity. The broadband optical/dielectric properties of the phospholipid bilayer in the frequency range from terahertz to mid-infrared with subnanometer resolution are still unclear.

To verify whether the myelinated nerve fiber is an applicable candidate for terahertz/mid-infrared electromagnetic propagation, herein, we theoretically investigate the spatially resolved dielectric properties of the phospholipid bilayer broadly ranging from 1 to 100 THz *via* molecular dynamics (MD) simulations for the first time. A membrane-water system based on DMPC molecules is constructed, and the space-frequency distribution of the dielectric properties in terms of the equilibrated membrane-water system is computed. The optimal band for electromagnetic propagation through the myelinated nerve fiber is discussed, and the real and imaginary permittivities across the equilibrated membrane-water system in this band are presented.

## Materials and methods

### Material characteristics

For any homogeneous, linear and isotropic biomaterial, its optical constant $\tilde{n}$ and dielectric constant $\tilde{\varepsilon }$ are frequency-dependent and can be expressed as follows:

$$\begin{array}{c} \tilde{n}=n\left(\omega \right)+i\kappa \left(\omega \right) \end{array}$$ (1)

$$\begin{array}{c} \tilde{\varepsilon }=\varepsilon \prime \left(\omega \right)+i\varepsilon^{\prime \prime }\left(\omega \right) \end{array}$$ (2)

where n(ω) and κ(ω) are respectively the refractive index and the extinction coefficient at angular frequency ω , ε′(ω) and $\varepsilon^{\prime \prime }\left(\omega \right)$ are respectively the real and imaginary parts of the permittivity at angular frequency ω , and i is the imaginary unit. If the magnetic properties of the biomaterial can be neglected, according to the physical definitions of the optical and dielectric constants, the following relation can be easily derived

$$\begin{array}{c} {\tilde{n}}^{2}=\tilde{\varepsilon } \end{array}$$ (3)

Substituting Eq. 1 and Eq. 2 into Eq. 3, we can obtain that

$$\begin{array}{c} \varepsilon^{\prime }\left(\omega \right)=n^{2}\left(\omega \right)-\kappa^{2}\left(\omega \right) \end{array}$$ (4)

$$\begin{array}{c} \varepsilon^{\prime \prime }\left(\omega \right)=2n\left(\omega \right)\kappa \left(\omega \right) \end{array}$$ (5)

Eq. 4 and Eq. 5 give rigorous mathematical relations between the optical constant and the dielectric constant. The values of $\varepsilon^{\prime }\left(\omega \right)$ and $\varepsilon^{\prime \prime }\left(\omega \right)$ can be determined by the values of n(ω) and κ(ω) , and vice versa. Therefore, if we have obtained the frequency-dependent dielectric properties of the biomaterial, its frequency-dependent optical properties are also known to us. Moreover, the values of $\varepsilon^{\prime }\left(\omega \right)$ and $\varepsilon^{\prime \prime }\left(\omega \right)$ , or n(ω) and κ(ω) , are interrelated as well. In the case of $\varepsilon^{\prime }\left(\omega \right)$ and $\varepsilon^{\prime \prime }\left(\omega \right)$ , Kramers–Kronig relations establish their relations as follows (Bertie and Zhang, 1992):

$$\begin{array}{c} \varepsilon^{\prime }\left(\omega \right)=\varepsilon_{\infty }+\frac{2}{\pi }P\int_{0}^{\infty }\frac{\omega^{\prime }\varepsilon^{\prime \prime }\left(\omega^{\prime }\right)}{{\left(\omega^{\prime }\right)}^{2}-\omega^{2}}d\omega^{\prime } \end{array}$$ (6)

$$\begin{array}{c} \varepsilon^{\prime \prime }\left(\omega \right)=-\frac{2}{\pi }P\int_{0}^{\infty }\frac{\omega \varepsilon^{\prime }\left(\omega^{\prime }\right)}{{\left(\omega^{\prime }\right)}^{2}-\omega^{2}}d\omega^{\prime } \end{array}$$ (7)

where $\varepsilon_{\infty }$ is the optical dielectric constant equal to the real part of the permittivity at infinite frequency and the symbol P denotes the Cauchy principal value which indicates that the integration range is cut open at the point where the integrand is singular. The relations of n(ω) and κ(ω) show the similar expressions as Eq. 6 and Eq. 7 and will not be listed here.

Phospholipids, the main components in biological membranes, are a sort of important biomaterial. A phospholipid molecule is basically composed of carbon, hydrogen, oxygen, nitrogen and phosphorus, and should be divided into two parts according to the different chemical characteristics: the hydrophilic head and the hydrophobic tail. The hydrophilic head includes the polar phosphatidylcholine while the hydrophobic tail includes the non-polar aliphatic chain. For this reason, under liquid condition phospholipids exist in the form of the double layer structure with their hydrophilic heads outside and hydrophobic tails inside, which is known as the phospholipid bilayer. Related studies have confirmed that biological membranes often in the liquid crystalline state in order to maximize their functional roles (Molugu et al., 2017; Paracini et al., 2018). Most naturally occurring phospholipids (such as DMPC, DPPC, etc.) behave slight differences in the length of the hydrophobic tails (Guo et al., 2014), and thus their optical and dielectric properties should vary little. Herein, we use DMPC for our study as the mammalian membranes contain abundant amounts of this type of phospholipid (Jurczak et al., 2021). It should be noted that the DMPC membrane has a phase transition temperature about 23–24°C, meaning that the membrane is in the gel (liquid crystalline) state exhibiting immobile (fluidic) feature below (above) this temperature (Kučerka et al., 2011).

### Model and MD simulation

**FIGURE 1:**
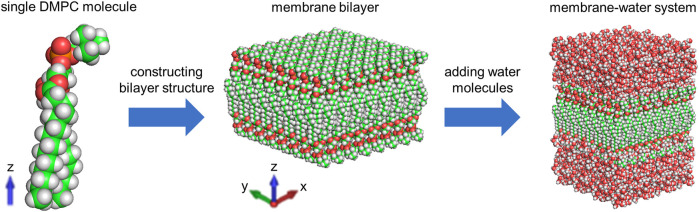
Model of the membrane-water system based on DMPC molecules for the MD simulation.

MD simulation is a powerful technique to understand the physical basis of the structure as well as the function of biomacromolecules (Karplus and McCammon, 2002). It is applicable to simulating the dynamic motions of a number of biological systems at atomic or near-atomic level of detail (Brandman et al., 2012; Zhao et al., 2013). Herein, we perform the MD simulation with GROMACS, which is one of the most widely used MD package (Abraham et al., 2015). As illustrated in Figure 1, we place a single DMPC molecule along the *z* direction and use it as the smallest unit. A membrane bilayer is then built by replicating this smallest unit in both *x* and *y* directions and inverting in the *z* direction. The completed membrane bilayer is composed of 128 DMPC molecules with 64 per leaflet, orienting parallel to the *x*-*y* plane with the normal in the *z* direction. The equivalent area per DMPC molecule is 0.606 nm, which is consistent with the experimental values (Wohlert and Edholm, 2006). The distance between two phosphorus atoms which are inverted relative to each other is 3.3 nm. Finally, a membrane-water system is constructed by adding 6,968 water molecules on both sides of the membrane bilayer to well hydrate it, which satisfies the actual state of biological membranes (Park et al., 2020). The dimension of the membrane-water system is approximately 6.23 nm × 6.23 nm × 9 nm.

Before conducting the MD simulation, it is worth noting that the periodic boundary condition is used to avoid edge effects owing to the finite size of the constructed membrane-water system. In addition, the all-atom CHARMM36 force field and the TIP3P water model are used to achieve high-accuracy computation, and the Particle Mesh Ewald method is used for high-efficiency computation of the long-range electrostatic interactions. We keep the simulation temperature at 310 K which is above the phase transition temperature of the DMPC membrane by applying Nosé-Hoover thermostat algorithm. After energy minimization, NVT and NPT ensembles, an equilibrium system can be established and its equilibrated data of the atoms such as charges, velocities and positions can be collected for post-processing.

The frequency range of the spectrum computed by GROMACS is determined based on the sampling theorem as

$$\begin{array}{c} \frac{2}{T}\ll f\ll \frac{1}{2\text{d}t} \end{array}$$ (8)

where f=ω/2π is the frequency, T is the length of the simulation time and dt  is the timestep. It can be inferred from Eq. 8 that the frequency resolution of the computed spectrum should be 2/T . Theoretically, a longer simulation time can result in a more accurate spectrum. However, as the simulation time increases the computational burden should be aggravated dramatically, and hence a compromise between the simulation accuracy and cost is inevitable. Herein, we set the values of T and dt to be respectively 1 ns and 1.5 fs in our simulation to investigate terahertz to mid-infrared dielectric spectra of the phospholipid bilayer.

### Subnanometer-scale dielectric spectra computation

We attempt to slice the equilibrium system along the *z* direction into multi-layered structures with subnanometer resolution and compute the dielectric spectra for each layer which can be treated as a homogeneous, linear and isotropic material. Based on the linear response theory (Iftimie and Tuckerman, 2005), the following relation can be derived for each layer

$$\begin{array}{c} n\left(\omega \right)\alpha \left(\omega \right)=\frac{1}{6c\varepsilon_{0}Vk_{B}T}\int_{-\infty }^{\infty }dte^{-\text{i}\omega t}\frac{\mathrm{dM}\left(0\right)}{dt}\cdot \frac{\mathrm{dM}\left(t\right)}{dt} \end{array}$$ (9)

where α(ω) is the absorption coefficient at angular frequency ω which is equal to 2κ(ω)ω/c , c is the velocity of light, $\varepsilon_{0}$ is the vacuum permittivity, V is the volume, $k_{B}$ is Boltzmann constant and T is the temperature. The angular brackets represent an ensemble average taken over all time origins and dM(0)/dt  and dM(t)/dt  are respectively the time derivatives of the total dipole moment at times 0 and t . According to the physical definition of the dipole moment, it can be easily derived that

$$\begin{array}{c} \frac{\mathrm{dM}\left(0\right)}{dt}\cdot \frac{\mathrm{dM}\left(t\right)}{dt}=\sum_{j=1}^{N}q_{j}v_{j}\left(0\right)\cdot \sum_{k=1}^{N}q_{k}v_{k}\left(t\right) \end{array}$$ (10)

where N is the number of all atoms, $q_{j}$ is the charge of *j*th atom, $q_{k}$ is the charge of *k*th atom, $v_{j}\left(0\right)$ is the velocity of *j*th atom at time 0 and $v_{k}\left(t\right)$ is the velocity of *k*th atom at time t . We note that the right-hand side of this equation represents the electrical flux-flux correlation function, which can be directly computed using the equilibrated data generated by the MD simulation. Substituting Eq. 5 and Eq. 10 into Eq. 9, we can obtain that

$$\begin{array}{c} \varepsilon^{\prime \prime }\left(\omega \right)=\frac{1}{6\varepsilon_{0}Vk_{B}T\omega }\int_{-\infty }^{\infty }\text{d}te^{-\text{i}\omega t}\sum_{j=1}^{N}q_{j}v_{j}\left(0\right)\cdot \sum_{k=1}^{N}q_{k}v_{k}\left(t\right) \end{array}$$ (11)

After the imaginary part of the frequency-dependent permittivity for each layer is computed, the real part $\varepsilon^{\prime }\left(\omega \right)$ can be obtained by using Eq. 6 and then the subnanometer resolution dielectric spectra of the phospholipid bilayer are determined.

### Optical dielectric constant estimation

We can see from Kramers–Kronig relations that it is enough to know $\varepsilon^{\prime }\left(\omega \right)$ to determine $\varepsilon^{\prime \prime }\left(\omega \right)$ while insufficient to compute $\varepsilon^{\prime }\left(\omega \right)$ just by $\varepsilon^{\prime \prime }\left(\omega \right)$ due to the existence of the unknown optical dielectric constant $\varepsilon_{\infty }$ . Actually, for transparent materials such as the phospholipid molecule their vibrational and electronic absorptions are both very weak in the range from near-infrared to near-ultraviolet, and consequently no optical absorption can be seen in their extinction coefficient spectra and the corresponding refractive index spectra demonstrate a feature of the normal dispersion. In this frequency range, the refractive index varies little and can be used to estimate the value of $\varepsilon_{\infty }$ . Based on Cauchy dispersion model (Cauchy 1830), the optical constant of a phospholipid molecule from near-infrared to near-ultraviolet region can be well described as

$$\begin{array}{c} n\left(\lambda \right)=A+\frac{B}{\lambda^{2}}+\frac{C}{\lambda^{4}} \end{array}$$ (12)

$$\begin{array}{c} \kappa \left(\lambda \right)=0 \end{array}$$ (13)

where n(λ) and κ(λ) are the refractive index and the extinction coefficient at wavelength λ , respectively. The parameters A , B and C are fit coefficients. It can be found that A is dimensionless and mainly contributes to the near-infrared region of the refractive index spectrum. Theoretically, n(λ) tends to be A when λ approaches infinity. The units of B and C are nm^2^ and nm^4^, respectively. These two parameters contribute to the visible and near-ultraviolet regions of the refractive index spectrum, respectively.

In order to determine Cauchy dispersion model of the phospholipid molecule, we need to know the values of its fit coefficients. It can be easily solved if we know three pairs of values of wavelength and refractive index. Herein, we use Vogel method and Lorentz-Lorenz equation to achieve this (Vogel 1948; Cao et al., 2009). Based on Vogel method, a molecule under investigation is divided into many chemical units (atoms, structures and groups) with known molar refractions at 468.3, 589.3 and 653.3 nm. By adding up the molar refractions of all the chemical units, the molar refractions of a molecule at these three wavelengths are obtained, and the corresponding refractive indexes are calculated using Lorentz-Lorenz equation:

$$\begin{array}{c} n\left(\lambda \right)=\sqrt{\frac{M+2\rho \cdot R\left(\lambda \right)}{M-\rho \cdot R\left(\lambda \right)}} \end{array}$$ (14)

where R(λ) is the molar refraction at wavelength λ , M is the molecular weight and ρ is the density. At this point we have known three pairs of values of wavelength and refractive index, and substituting these values into Eq. 12 we can compute the values of the fit coefficients in Cauchy dispersion model.

**FIGURE 2:**
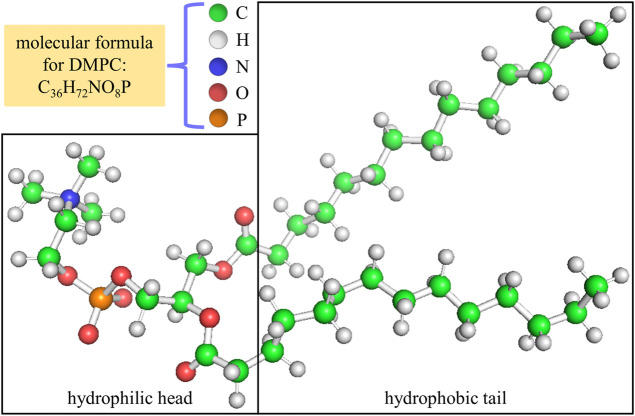
Position of the dividing line between the hydrophilic head and the hydrophobic tail in a DMPC molecule.

Generally speaking, it is expected that the dielectric properties for the hydrophilic head and the hydrophobic tail of a phospholipid molecule should behave differently owing to their obvious difference in chemical constituent. Therefore, we separately calculate the optical dielectric constants of the hydrophilic head and the hydrophobic tail and use them as reference values for the optical dielectric constants of all sublayers in the hydrophilic head and the hydrophobic tail. For a DMPC molecule, the dividing line between its hydrophilic head and hydrophobic tail is demonstrated in Figure 2. The chemical units used for the hydrophilic head and the hydrophobic tail are listed in Tables 1, 2.

**TABLE 1:** Chemical units used for the hydrophilic head of a DMPC molecule.

Chemical unit	Number	*R* (468.3 nm)	*R* (589.3 nm)	*R* (653.3 nm)
CH_3_	3	5.719	5.653	5.636
CH_2_	5	4.695	4.647	4.624
COO	2	6.261	6.200	6.173
C	1	2.601	2.591	2.572
H	1	1.043	1.028	1.026
PO_4_	1	10.821	10.769	10.733
N	1	2.820	2.744	2.698

**TABLE 2:** Chemical units used for the hydrophobic tail of a DMPC molecule.

Chemical unit	Number	*R* (468.3 nm)	*R* (589.3 nm)	*R* (653.3 nm)
CH_3_	2	5.719	5.653	5.636
CH_2_	23	4.695	4.647	4.624

## Results and discussion

**FIGURE 3:**
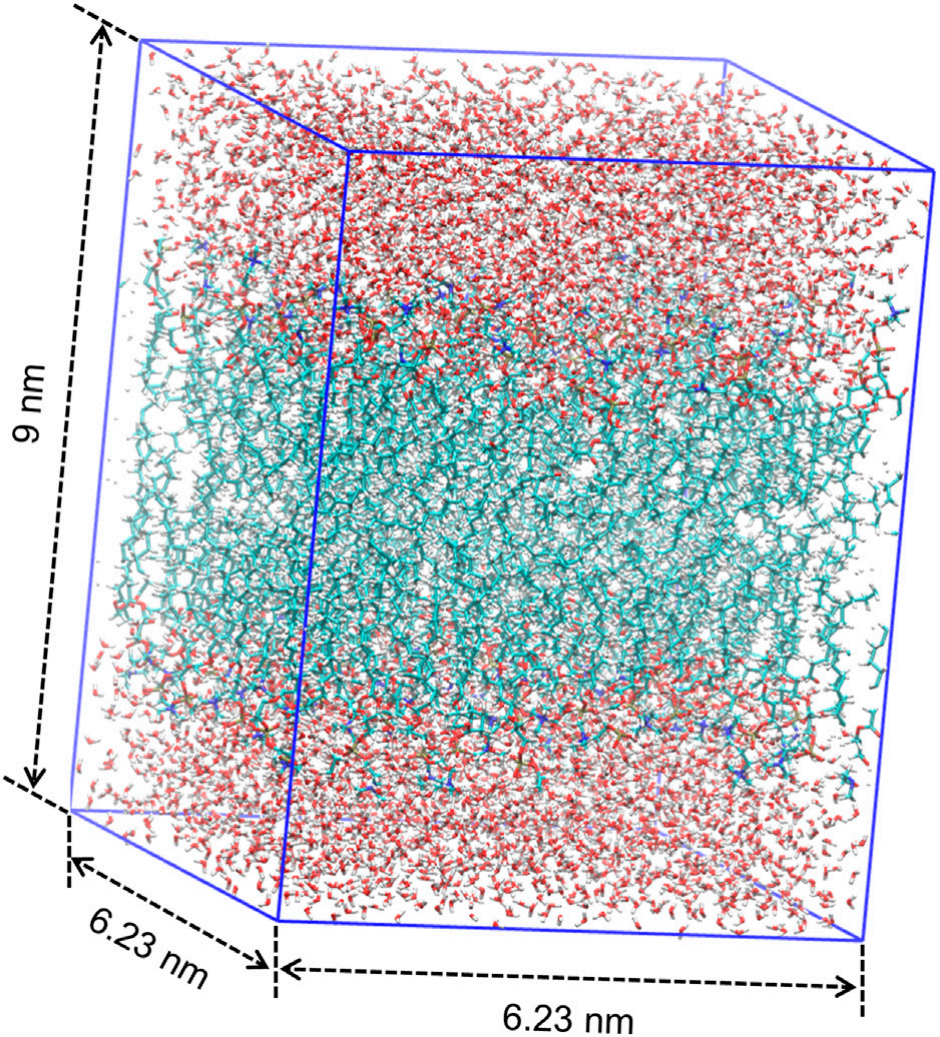
Final structure of the equilibrated membrane-water system.

**FIGURE 4:**
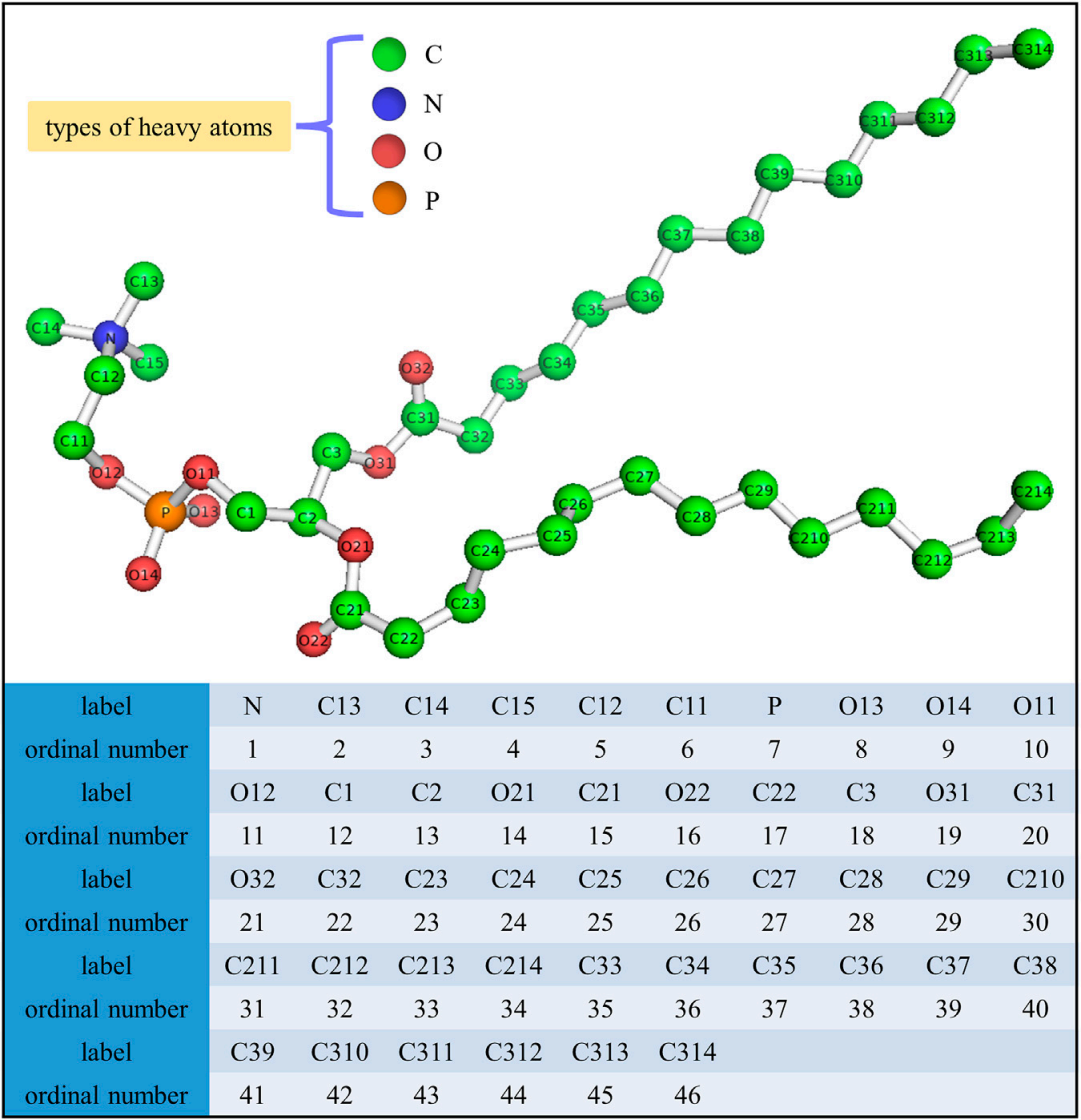
Label and the corresponding ordinal number for each heavy atom in a DMPC molecule.

**FIGURE 5:**
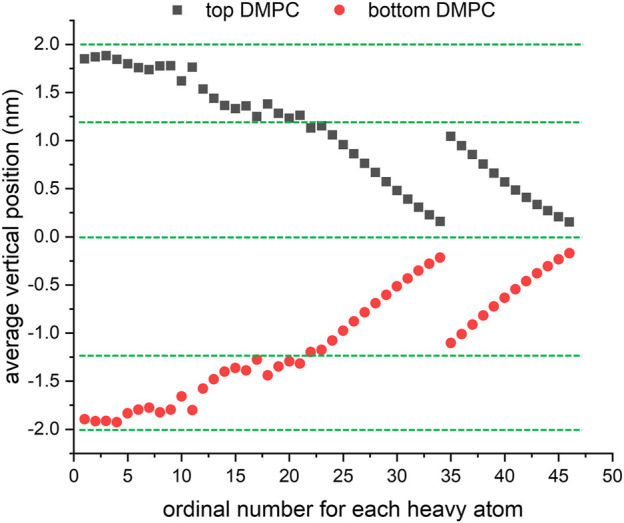
Average vertical position versus ordinal number for each heavy atom in the equilibrated DMPC bilayer.

**FIGURE 6:**
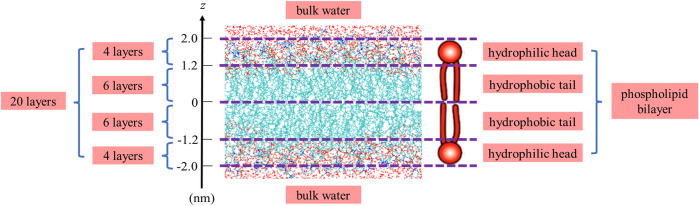
Details of layering for the equilibrium system.

The final structure of the equilibrated membrane-water system is displayed in Figure 3, where we can find that the hydrophobic tails of all phospholipid molecules are contracted as well as disordered. Herein, the equilibrium system is layered based on the average vertical position of each heavy atom (non-hydrogen atom) in the phospholipid bilayer. In total, there are 46 heavy atoms in a DMPC molecule. We label these atoms as shown in Figure 4, and then assign each label a specific ordinal number. Figure 5 gives the result of the average vertical position versus ordinal number for each heavy atom in the equilibrated DMPC bilayer. It is noted that for the pair of heavy atoms with the same ordinal number which are respectively located at the top and bottom DMPC molecules, their average vertical positions are substantially symmetric about the plane of *z* = 0. The thickness of the DMPC monolayer is approximately 2 nm, with 0.8 nm for the hydrophilic head and 1.2 nm for the hydrophobic tail. In order to study the subnanometer resolution dielectric properties of the biomembrane, we use 0.2 nm thickness as spacing of layers and finally slice the phospholipid bilayer into 20 layers, as shown in Figure 6.

**FIGURE 7:**
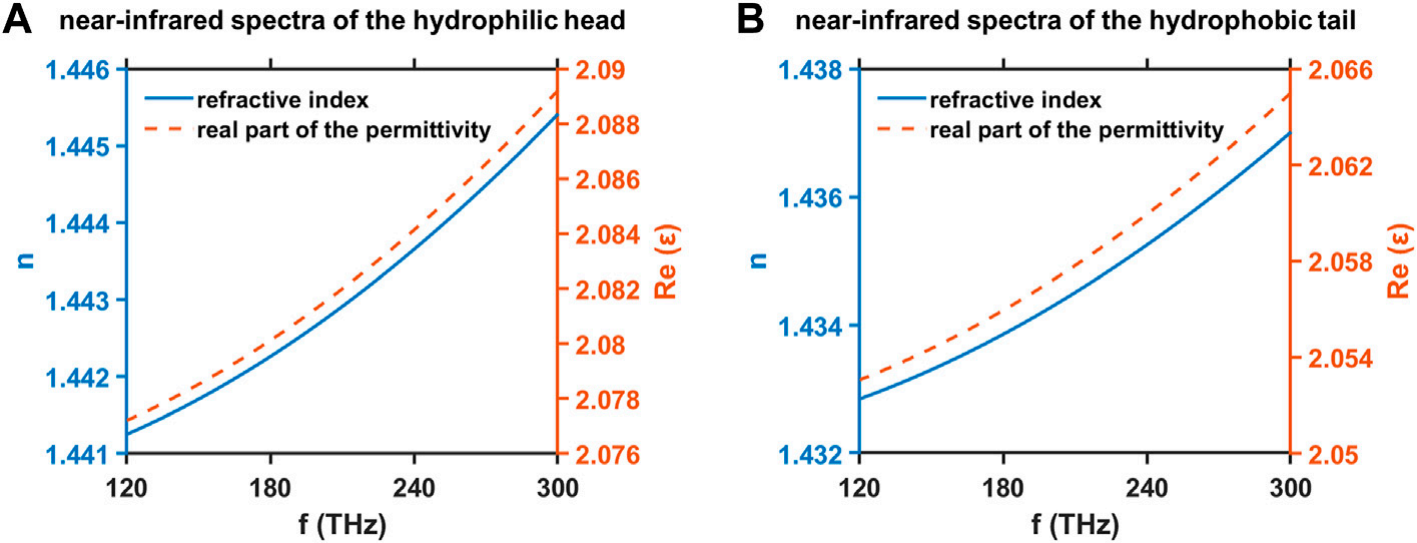
Refractive index n and real part of the permittivity ε′ at near-infrared frequency f for the hydrophilic head **(A)** and the hydrophobic tail **(B)** of a DMPC molecule through the Cauchy dispersion model and the Vogel method.

We conduct the theoretical estimation of the refractive index n (real part of the permittivity ε′ ) for the hydrophilic head and the hydrophobic tail of a DMPC molecule through Cauchy dispersion model as well as Vogel method, and the corresponding near-infrared spectra are shown in Figure 7. For both the hydrophilic head and the hydrophobic tail, their n and ε′ vary little in the near-infrared region, and the hydrophilic head has higher amplitudes than the hydrophobic tail. Herein, the values of ε′ at 300 THz for the hydrophilic head and the hydrophobic tail are adopt as the optical dielectric constants of all sublayers in the hydrophilic head and the hydrophobic tail, respectively.

**FIGURE 8:**
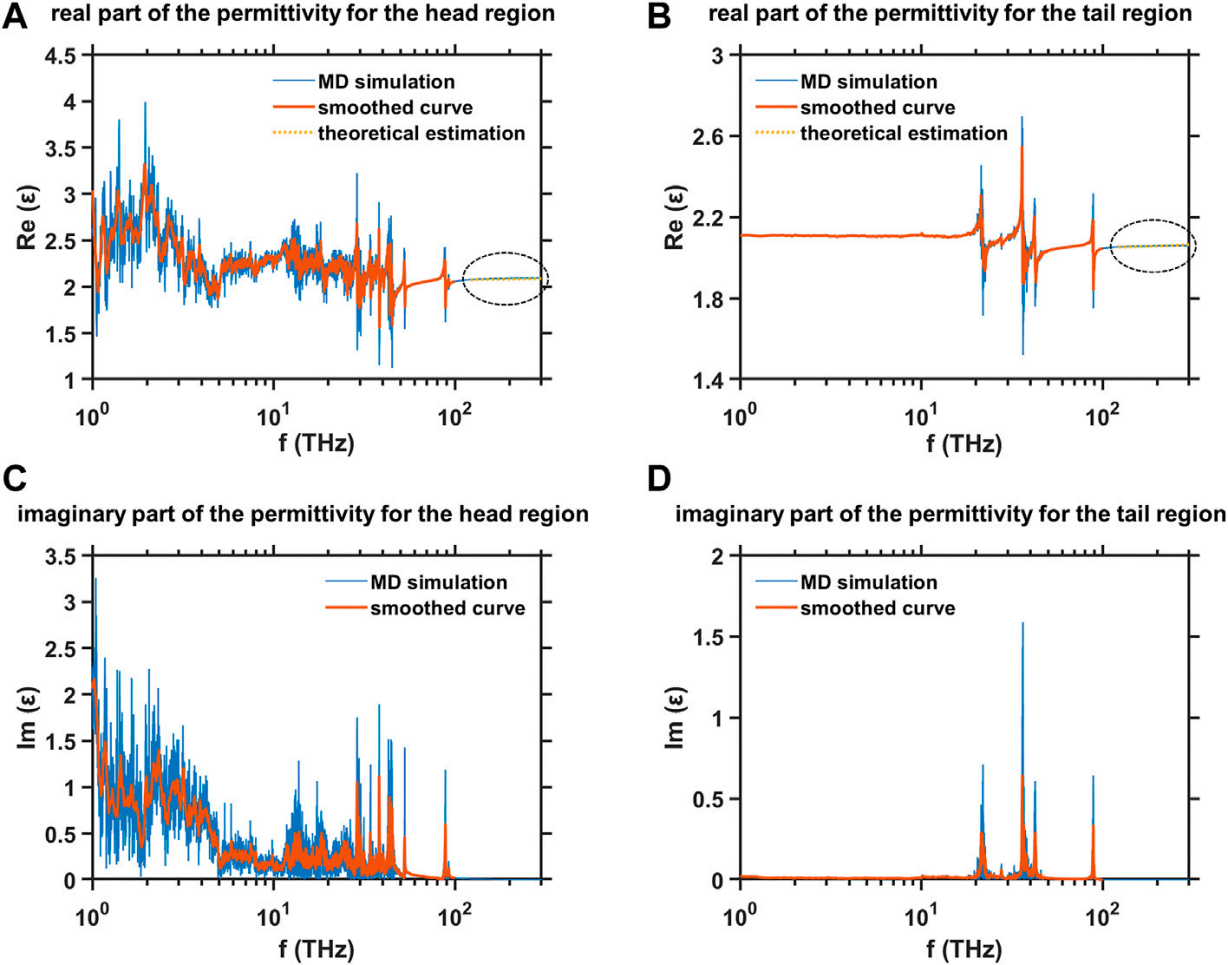
MD simulation results of the dielectric spectra in the frequency range from terahertz to mid-infrared for two regions of hydrophilic head and the hydrophobic tail in the equilibrated DMPC monolayer **(A)** Real part of the permittivity for the head region, **(B)** real part of the permittivity for the tail region, **(C)** imaginary part of the permittivity for the head region, and **(D)** imaginary part of the permittivity for the tail region.

The dielectric spectra in the frequency range from terahertz to mid-infrared for two regions of the hydrophilic head and the hydrophobic tail in the equilibrated DMPC monolayer are studied by MD simulation, and the results are shown in Figure 8. The original data is smoothed based on the moving average window to offer the readers a clearer view of the variation trends of the dielectric spectra. The imaginary parts of the permittivity $\varepsilon^{\prime \prime }$ for the head and tail regions are first computed, and the real parts ε′ are then obtained through Kramers–Kronig relations. It should be noted that the near-infrared data are also included in the spectra which agree well with the results of theoretical estimation, indicating the validity of MD simulation. In most cases, both the values of ε′ and $\varepsilon^{\prime \prime }$ for the head region are higher than those for the tail region, and only at some limited frequencies the head region shows lower amplitudes. This is mainly caused by the high polarity of the head region. In addition, it can be seen that there are many characteristic peaks in the spectra, which is due to the effects of the chemical bond vibration such as C-H stretching and COO stretching.

**FIGURE 9:**
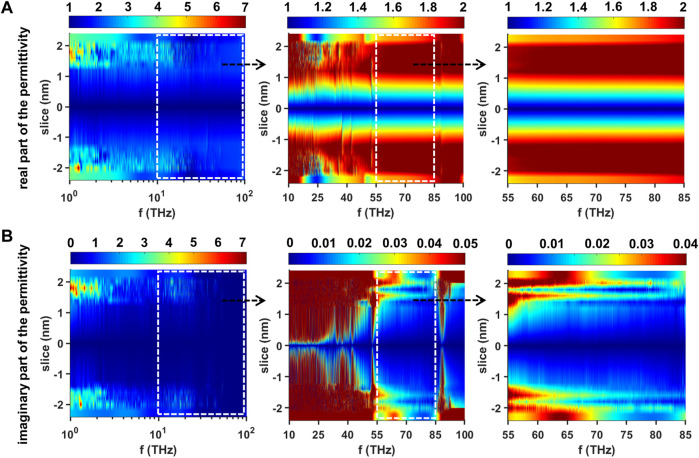
Dielectric spectra of the equilibrated membrane-water system in the range of 1–100 THz **(A)** Real part of the permittivity in the range of 1–100 THz (left), 10–100 THz (middle) and 55–85 THz (right) and **(B)** imaginary part of the permittivity in the range of 1–100 THz (left), 10–100 THz (middle) and 55–85 THz (right). The dashed rectangles are used to outline the regions we are interested. Each region is further enlarged and redrawn in its right adjacent subfigure.

**FIGURE 10:**
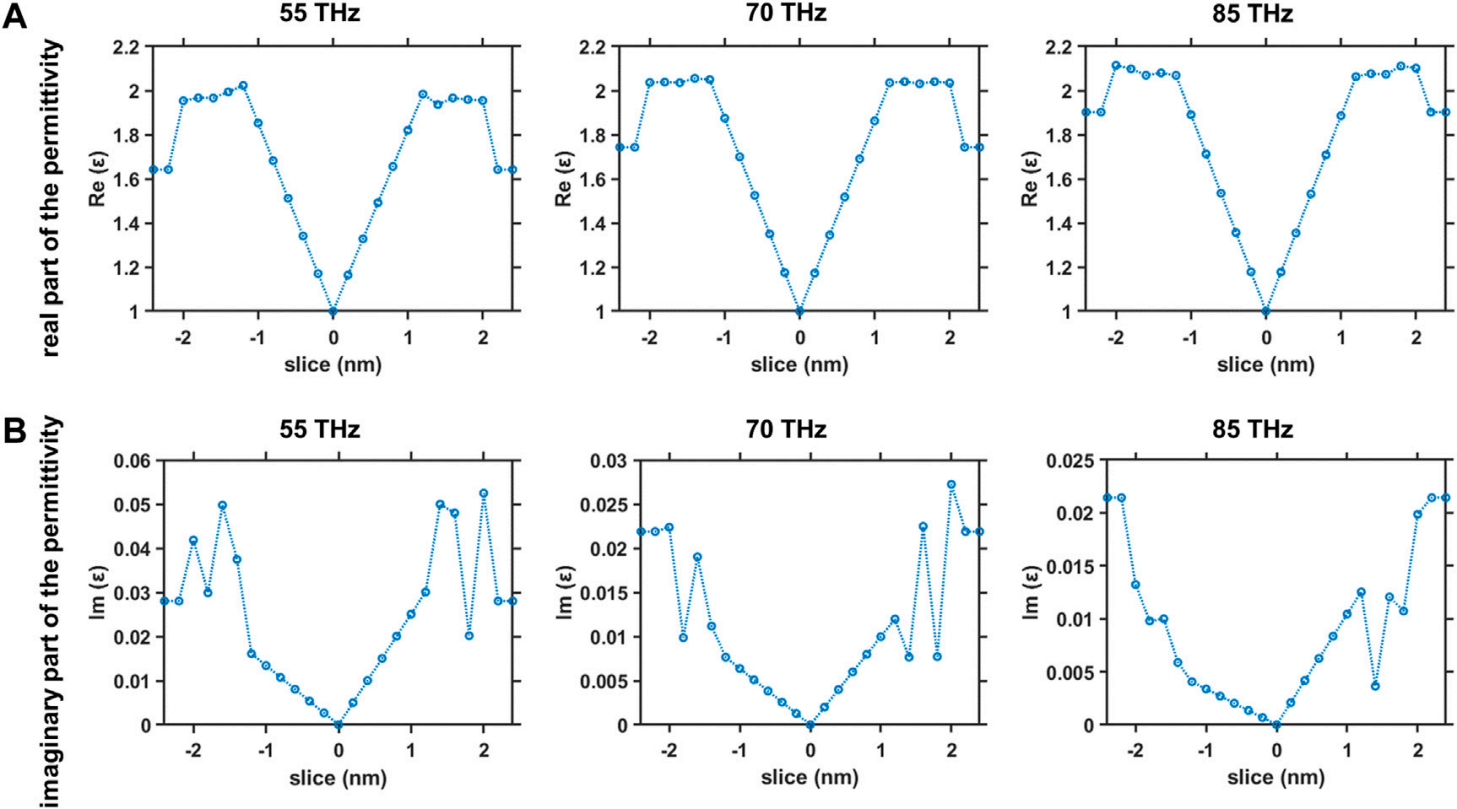
Dielectric properties across the equilibrated membrane-water system at frequencies in the range of 55–85 THz **(A)** Real part of the permittivity versus slice position at 55 THz (left), 70 THz (middle) and 85 THz (right) and **(B)** imaginary part of the permittivity versus slice position at 55 THz (left), 70 THz (middle) and 85 THz (right).

Results of the subnanometer resolution dielectric spectra in the frequency range from terahertz to mid-infrared (1–100 THz) in terms of the equilibrated membrane-water system are shown in Figure 9. The layered data have been further interpolated to get a smooth dielectric spectrum landscape. We can obviously see that the dielectric properties of the equilibrated phospholipid bilayer are also substantially symmetric about the plane in which the vertical position of the slice is 0. In the range of 1–10 THz, the values of ε′ of the head region can be up to about 7 at some specific slice positions and frequencies, while in these cases the corresponding values of $\varepsilon^{\prime \prime }$ are also very large. Furthermore, the characteristic peaks of the head and tail region can be found to be mainly concentrated in the range of 1–55 THz. Overall, the frequency range of 1–55 THz might not be an ideal band for electromagnetic propagation through the myelinated nerve fiber. This band not only cannot meet the requirements of continuity and width for robust electromagnetic propagation, but also cannot support the long-distance electromagnetic propagation due to the severe attenuation caused by the large values of $\varepsilon^{\prime \prime }$ . In addition, when the frequency is below 20 THz or above 85 THz, the values of $\varepsilon^{\prime }$ of the head region can be lower than those of the water region, which can lead to the electromagnetic waves spreading to the region outside the myelinated nerve fiber. Actually, the band of 55–85 THz can be a good frequency window in which all the conditions for electromagnetic propagation within the myelin sheath are satisfied. Figure 10 shows the dielectric properties across the equilibrated membrane-water system at different frequencies chosen from the range of 55–85 THz. We can clearly see that the values of $\varepsilon^{\prime }$ at the head layers are larger than those at the tail and water layers, and the values of $\varepsilon^{\prime \prime }$ at both the head and tail layers are trivial. Therefore, it can be inferred that the electromagnetic waves working at this band mainly propagate along the head regions of the phospholipid bilayer of the myelinated nerve fiber.

## Conclusion

In this work, to explore the possibilities of the myelinated nerve fiber acting as a terahertz/mid-infrared dielectric waveguide, we for the first time construct the spatially sub-nm resolved dielectric spectrum in a frequency band widely ranging from 1 to 100 THz regarding a membrane-water system, where the phospholipid bilayer is the major constituent of the myelin sheath. According to calculations on roughly divided head and tail regions of the bilayer, we find that the head region shows higher values of $\varepsilon^{\prime }$ and $\varepsilon^{\prime \prime }$ compared with the tail region in the majority of the band. After a fine layering, the membrane-water system is further analyzed in terms of the space-frequency dielectric distribution. Clearly, the head layers possess larger $\varepsilon^{\prime }$ values than those of the tail and water layers in 55–85 THz, while the $\varepsilon^{\prime \prime }$ values at both the head and tail layers are trivial. This suggests a long-distance electromagnetic propagation within the myelin sheath especially the head regions of the phospholipid bilayer in this specific band. Our finding could be a theoretical evidence that the mid-infrared electromagnetic waves could serve as a type of highly stable information carrier for neural signal propagation.

## Data Availability

The original contributions presented in the study are included in the article/Supplementary Material, further inquiries can be directed to the corresponding author.
